# Is there a doctor in the house? Availability of Israeli physicians to the workforce

**DOI:** 10.1186/s13584-017-0157-0

**Published:** 2017-05-30

**Authors:** Pamela Kuflik Horowitz, Annarosa Anat Shemesh, Tuvia Horev

**Affiliations:** 1 Administration for Strategic and Economic Planning, Ministry of Health, 39 Yirmiyahu Street, Jerusalem, Israel; 2 Department of Health Systems Management, Guilford Glazer Faculty of Business and Management, Ben-Gurion University of the Negev, Beersheba, Israel

**Keywords:** Physician outflow, Workforce planning, Physician work-hours, Physician shortage

## Abstract

**Background:**

Israeli policymakers have expressed serious concerns about being able to meet the growing demand for physician services. For this reason, the Israel Ministry of Health (MoH) undertook studies based on 2008 and then 2012 data to obtain an accurate assessment of the size, specialty mix, demographic and geographic composition of the physician workforce. This paper highlights the findings from these studies about the number and percentage of licensed physicians in Israel who were not available, were only partially available, or were about to leave the Israeli healthcare workforce.

**Methods:**

The two studies cross-linked administrative files of the entire physician population in Israel. The two sources were the MoH registry of licensed physicians, which contains demographic, medical education and specialty information, and the Israel Tax Authority income file on employment data. A third source, used only for the study of 2008 data, was the CBS Population Census Data 2008 which was based on a large representative sample of the population (14%), along with the updated Population Registry, which provided data on physicians whose occupation was in medical care as well as the number of work-hours. By linking the files we could also assess the population of licensed Israeli physicians living abroad.

**Results:**

Only 74% of licensed physicians of all ages in 2012 were active in the Israeli workforce. Of physicians under the age of 70, 87% were living and working in Israel. Female physicians tended to retire from the workforce earlier than males and were more likely to work fewer hours during their working years. The rate of physicians who worked longer hours declined in both genders as age rose. About 10% of licensees had been living abroad for at least a year and the majority of these were older. Approximately 7% of licensed physicians, ages 30–44, were abroad and most are presumed to be doing additional clinical training or gaining research experience.

In some specialty fields young physicians were not replacing retirees at a compensatory rate; anesthesiologists, a specialty in short supply in Israel were more likely to be living abroad than other specialists.

**Conclusions:**

Assessment of the medical workforce pool and personnel planning require not just the number of licensed physicians but also information about the employment mix of license holders and their level of professional activity in Israel.

For planning future workforce needs, it is important to keep in mind that the average female vs. male physician has lower clinical productivity due to shorter hours and earlier retirement and that a group of young physicians will predictably be abroad at any point in time; however major “brain drain” is not evident. Furthermore, extrapolating from the findings in the current studies, we believe that a potential shortage of physicians within Israel can be mitigated by better administrative support of physicians, use of physician extenders, and careful attention to improving physician satisfaction in certain specialties.

## Background

In order to ensure the appropriate and efficient delivery of quality health care in a country, an adequate supply of physicians is needed to match the demand for services. In recent decades policy-makers in many countries around the world, Israel among them, have raised concerns that a variety of social, demographic, technological and economic factors, will lead to insufficient physician supply in the coming years [1].

As aptly stated in a working paper from the Organization for Economic Co- operation and Development (OECD) on health workforce planning, there is a "need to know first where we are, before we can know where we're heading: The first step of any good health workforce projection is good data about the current situation" [2].

That was the goal of an Israeli study based on 2008 data [3]. The study aimed at obtaining an accurate as possible head count of all licensed physicians who were practicing medicine in Israel including demographic, employment and geographic characteristics.

In Israel, physicians are licensed just once in their professional lifetimes, and past assessments of physician supply were primarily based on the Ministry of Health (MoH) registry of all licensed physicians. The physician registry, although updated according to the population registry for deaths, does not include employment status information. In the past a number of attempts were made by the MoH to acquire more complete and accurate information on employment status [4, 5]. The Central Bureau of Statistics (CBS) also regularly publishes reports on healthcare personnel based on their annual labor force surveys. Since 2010, the MoH added to this data base by approaching most healthcare service providers (HMO’s and hospitals) for information on their employees [6]. Those sources, while important, are insufficient, being based on either sample surveys or providing only partial information.

In the present paper, the authors, who were part of the original team of the 2008 and 2012 studies, wish to highlight new findings from the 2012 administrative data [7] and the CBS Population Census Data 2008. These findings focus on physician outflow from the Israeli medical system, information that is crucial to the process of personnel projection. Outflow includes those who were either partially inactive or completely unavailable to the workforce, such as physicians who were licensed to practice medicine but who were not working at all, those who were living abroad for at least 1 year, those who worked only part-time, those who were nearing retirement age and thus about to leave the workforce and those who abandoned the practice of medicine for other professions.

## Methods

In the study based on the 2008 data, national administrative files were cross-linked to obtain figures on the active and inactive medical workforce and its demographic and geographic distributions. In 2012 the study was repeated using the same methodology. Both studies derived data from two sources in their respective years, and the study using 2008 data also drew on the most recent (2008) CBS Population Census, specifically its Socio-Economic File (SEF). The two sources included in both analyses were the MoH registry of licensed physicians, which contains demographic, medical education and specialty information, and the Israel Tax Authority income file. The CBS receives the tax authority income file annually and it includes employment data that meet the Statistics’ Ordinance requirements for privacy. The SEF Population Census (2008) along with the updated Population Registry, provided data on physicians working in Israel whose occupation was in medical care, as well as the number of work-hours based on a large representative sample of the population (14%). In this paper, the 2012 data are presented whenever available and the 2008 data are presented when no updated data is available. The source of the data for each table is presented with each table.

By cross-linking the MoH physician registry and the updated Population Registry, data were also obtained on physicians who resided abroad (based upon the United Nations definition of permanent place of residence). These include anyone who left the borders of Israel for at least 12 months with only an allowance of visiting the homeland for not more than 90 days [8].

Since the Tax Authority Income file used by the CBS includes only data on civilian workers, we excluded from the study physicians who are practicing in a medical capacity in the armed forces.

## Results

### Number of physicians

The findings from the 2012 data pointed to a considerable gap between the numbers of licensed physicians listed in the MoH registry (32,775) and the number of physicians actually living and working in healthcare in Israel (24,261) according to the Tax Authority income file. The personal identity information did not permit data linkage for about 1% of the physicians in the MoH listings who were excluded from the study. About 10% of the licensed physician population was living abroad. Of the remaining 29,129 physicians living in Israel, almost 5000 fewer (24,261) were actively employed in Israel. Thus, of the total number of physicians registered with the MoH of all ages, only 74% were living and employed in Israel in 2012. The number of physicians under the age of 70 working in Israel in the health field in 2012 stood at 22,082. This was 87% of licensed physicians of that age who were living in Israel.

The percentages of licensed employed physicians aged 30–64, ranged from 92 to 94% and at age 65 the employment rate began, as expected, to decrease (Table 1). The percentage of women in the employed workforce peaked at 50% in the age group 30–34 and then declined gradually as age rose.

**Table 1:** Licensed and employed physicians in Israel and the percent of women from all employed physicians, by age (2012)

Age groups	Licensed physicians	Number employed in Israel	Percent employed	Percent of women
Total	29,129	24,261	83	40
To 29	818	665	81	45
30–34	2180	2021	93	50
35–39	2871	2639	92	46
40–44	2853	2614	92	47
45–49	3076	2890	94	45
50–54	3682	3469	94	41
55–59	3959	3697	93	38
60–64	3529	3205	91	34
65–69	2340	1893	81	27
70–74	1415	712	50	28
75–79	1018	315	31	23
80+	1388	141	10	14

The data were obtained by cross-linking the MoH physician registry and the Tax Authority income file

Table 2 displays the age and gender distribution of physicians in 2012. As shown, in the youngest age group, 12% (13% of younger men and 11% of younger women) were not employed.^1^ The middle age group (45–64) had the lowest non-employment rate (11%). A heightened difference in employment rates was found between the genders in the 65–69 age group with almost twice as many women as men not employed (37% and 20% respectively). Similarly, while 74% of the total population of physicians aged 70 and over was not employed, the rate for women was again much higher than that of men (85% vs. 65%). Female doctors tend to exit the workforce earlier in life than do male doctors. This may be partially explained by the earlier retirement age for women (62) than men (67).

**Table 2:** Licensed physicians living in Israel, not employed, by age group and gender (20l2)

	Total		Female		Male	
Age group	Number of licensed physicians	Percent not- employed	Number of licensed physicians	Percent not- employed	Number of licensed physicians	Percent not employed
Total	29,129	21	12,207	24	16,922	19
24–44	8722	12	4108	11	4614	13
45–64	14,246	11	5733	13	8513	10
65–69	2340	25	741	37	1599	20
65+	6161	55	2366	70	3795	46
70+	3821	74	1625	85	2196	65

The data were obtained by cross-linking the MoH physician registry and the Tax Authority income file

### Weekly work-hours

The overall rates of employment by age of male and female physicians do not reflect or account for the differences in hours worked per week. These can be viewed in Tables 3 and 4. Note that data on work hours was obtained from the 2008 census. The resulting weighted estimate for employed physicians actually working in medical care was 23,818 [3].

**Table 3:** Percent distribution of employed physicians by number of weekly work- hours, gender and age (2008 Census)

	Number of employed physicians	Number of weekly work-hours				
		1–20	21–40	41–60	61+	Unknown
Total physicians	23,818	5	26	45	15	9
Gender						
Male	14,072	4	19	47	19	11
Female	9724	6	36	41	10	7
Age group						
25–44	8127	2	19	44	24	11
45–64	13,668	2	28	49	12	9
65–69	1010	17	42	26	5	11
65+	2000	35	38	16	4	8
70+	990	53	33	6	3	6

The data were obtained by cross-linking the MoH physician registry and the Census data

**Table 4:** Percent of physicians who work less than full-time, by age and gender (2008 Census)

Age group	Total	Male	Female
25–44	21	12	31
45–64	30	21	46
65–69	58	48	82
65+	72	66	85
70+	85	85	88
Total	31	23	36

The data were obtained by cross-linking the MoH physician registry and the Census data

The cumulative number of weekly hours worked was self-reported by physicians for all places of work. The average total number of weekly work-hours was 48.4 h. Most employers in Israel define a full-time position for physicians as between 42 and 45 weekly hours. For our purposes, we will refer to those who work 40 h or fewer as those who work “less than full-time”.

Of the total population of working physicians in 2008, 59% were male and 41% were female. Almost twice as many women as men worked less than full-time (42% vs. 23%) (Table 3). Almost twice as many men (19%) as women (10%) worked more than 60 weekly hours, with a similar pattern seen in every age group (not shown). Overall, fewer younger physicians than older ones worked less than full-time, with the percentage of physicians who worked shorter hours rising with age. About a fifth of young physicians worked less than full-time compared to almost three quarters (73%) of those aged 65 and over (and 86% of those aged 70 and over).

Among younger physicians, over two-thirds worked more than 40 h a week as compared to 20% in the 65+ age group and 9% at 70 years and over.

It is interesting to note that only 5% of all employed physicians (of all ages) worked 20 h or less, and only 2% of those in each of the peak employment age categories (25–44, 45–64) did so (Table 3).

The hourly work distribution by age and gender (Table 4) indicated that working less- than-full-time rose with age for both genders, though the rate of women’s less-than-full-time employment was significantly higher in every age group until age 70 and upward, when the rates were very high for both genders. The study also found that in the 65 and over age group, three times as many male as female physicians worked more than 40 weekly hours (25% vs. 8%). No women at all recorded working 61 h or more past the age of 65 whereas 12% of men did so (not shown).

### Physicians living abroad

One of the largest groups of physicians unavailable to medical practice in Israel is that of licensed doctors residing abroad. In 2012 there were 3275 physicians (10% of total licensees) who had been living abroad for at least a year. Forty-five percent of those physicians were between the ages of 45 and 64 and 37% were aged 65 and over. Of licensed physicians aged 45–64, 10.4% lived abroad and of licensed physicians aged 65 and over, 20% did so. Almost twice as many male as female physicians (65% vs. 35%) lived abroad in 2012. From among the total population of physicians who resided abroad, 65% had received their medical degree abroad.

Ninety percent of those found to be living abroad in 2012 were there for at least 3 years and 82% were there for 5 years or more. An examination of the data by year in which the physicians were awarded his/her degree showed that as license-issue dates were earlier, the percent of physicians living abroad was higher (Table 5). The highest rate of physicians living abroad was among those whose license was issued before 1954 (22%) as compared to 5% of those who were awarded a degree between 2000 and 2012.

**Table 5:** Percent of physicians living abroad to age 69, by year of license issue (20l2)

Period of license issue	Number of licensed physicians	Percent living abroad
To 1954	144	22
1955–1969	1338	19
1970–1988	8418	14
1989–1999	10,244	10
2000–2012	8985	5

The data were obtained by cross-linking the MoH physician registry and the population registry

The distribution of medical specialists (by last specialty) who lived abroad and were thus unavailable to the healthcare system in Israel is shown in Table 6. This information can be assessed in relation to those fields of medicine that are in distress in Israel. For example, 15% of anesthesiologists resided abroad for at least a year, while at the same time anesthesiology is a field that is considered to be in shortage within Israel. On the other hand, relatively lower percentages of specialists in some other fields were living abroad in 2012 such as in: obstetrics (6%), family medicine (3%), child and adolescent psychiatry (3.5%) and negligible percentages of specialists in pediatric cardiology, pediatric surgery geriatrics and rehabilitation medicine.

**Table 6:** Rate of specialists from all Israeli specialists in selected fields, living abroad, to age 69 (20l2)

Field of specialty	Total Number of specialists	Living abroad (%)
Clinical microbiology/ Clinical chemistry	14	..
Anesthesiology	548	14.8
Pediatrics	1390	9.1
Psychiatry	953	7.6
General surgery	545	7.3
Pediatric intensive care	50	10.0
Pediatric cardiology	43	..
Oncology	168	8.3
Neonatology	135	5.2
Obstetrics/Gynecology	1154	5.9
Physiatry and Rehabilitation medicine	131	..
Family medicine	1647	3.0
Pediatric surgery	55	..
Geriatrics	283	..
Child and adolescent psychiatry	202	11.6

The data were obtained by cross-linking the MoH physician registry and the population registry

### Additional findings relating to workforce planning

The distribution of practicing specialists by age (Table 7) provides information that allows estimating the imminent retirement of specialists from practice and the scope of likely entry of young specialists into the respective specialty fields. In turn, one can estimate whether new, young specialists are likely to replace the retirees at a compensatory rate. For example, 24% of pediatric surgeons were age 65 and over in 2012, whereas, only 7% of pediatric surgeons were age 30–44.

**Table 7:** Percent of physicians working in Israel in selected specialties (last specialty), by age group (20l2)

Specialty area	Age groups			Number of specialists
	30–44	45–64	65+	
Pediatric surgery	7	69	24	59
Pediatric neurology	22	65	14	125
General surgery	20	65	14	498
Psychiatry	21	65	14	796
Pediatrics	35	57	8	1153
OB GYN	19	65	15	1060
Rehabilitation medicine	15	75	10	126
Child /adolescent psychiatry	24	64	12	177
Anesthesiology	19	70	11	454
Geriatrics	11	80	9	280
Neonatology	18	70	13	119
Internal medicine	42	51	8	1136
Intensive care	19	68	13	131
Family medicine	34	64	3	1448

The data were obtained by cross-linking the MoH physician registry and the population registry

The low percentage (3%) of family specialists that reached retirement age, together with the high percentage (34%) of young family specialists, is a reflection of the relative newness of family medicine as a specialty in Israel (since 1971).

## Discussion and recommendations

Unlike many other western countries,^2^ Israel does not have mandatory periodic re- registration for physicians, a mechanism which could serve to update and enhance knowledge of the employment status and patterns of the active workforce by collecting information about a physician’s activities at the time of renewal. Physician projection data has until now been largely based upon the number of registered medical licenses, information that does not take into account employment status, changes in residence, variations in retention rates and retirement patterns.

In years prior to this study, various professional bodies addressed the significance of the lack of accurate, updated data on the number and characteristics of employed physicians [9–13] and included among their recommendations taking steps to establish periodic re-registration for professionals in the health fields. Aside from improving the knowledge of active employment in the medical workforce, establishing physician re-registration in Israel would also likely add to the sense of commitment on the part of the physician to his or her clinical field of practice - all the more so if it also entailed continuing medical education requirements - and as such is recommended by the current authors.

The methodology upon which the current work was based [3, 7] took an important step forward towards obtaining accurate information on the gap between the physician registry and the actual, active physician workforce, as a starting point for workforce projection and planning. The study sheds light on inactive or only partially active license holders in Israel, on retirement patterns, and on age and gender differences within these parameters.

Studies have shown that female participation in the Israeli medical workforce has increased over time [5, 12–15], alongside a parallel increase in other OECD countries [16]. The female participation rate in Israel is higher than in the United States, where in 2012 about one-third of actively licensed physicians were women as compared to 41% in Israel [16]. The percentage of females in most specialties in Israel has also increased except in the field of anesthesiology, with the highest rates of females in family medicine followed by oncology, pediatrics and psychiatry [15]. By comparison, in the United States, the highest percentages of females specialists reported in a 2015 study were in obstetrics/gynecology, pediatrics, family medicine and psychiatry [17].

While the current study could not provide a precise number of work-hours per physician to convert to full-time equivalent (FTE) positions (one of the study’s limitations), the age-categorized data did demonstrate a lower work-hour contribution by female physicians. Data from other studies indicate that female physicians also practice at lower activity levels during childbearing age, have a higher tendency to work part time and see fewer patients per day. They also take more parental leave, are more likely to leave the practice of medicine during childbearing years and are more likely to retire early [1, 14, 15, 18, 19]. A study from Canada concluded that if current gender practice patterns of doctors persist "an overall decrease in doctor productivity is to be anticipated" [19].

Greater female workforce participation in Israel would mean fewer FTE positions to be contributed to the workforce [15], creating a situation described by the Horev "by which the supply of FTE physicians is rising at a slower rate as compared to the supply in terms of the number of active physicians" [13]. The trend of an increasing percentage of female new licenses seems however to have leveled off since 2006 where it was and remains at 41% [17]. Moreover, there is evidence that, in recent years the proportion of women among the younger physician population has been getting lower [3, 15].

The contribution of women to the physician workforce however is not solely a function of hours and years worked, but also of the kind of care and time given to patients, and the quality and nature of the professional relationship [15]. With the so-called ‘feminization’ of medicine, it has been hypothesized that there may be a shift towards a more bio-psychosocial approach to patient care changing both patient-doctor relationships and the nature of health care delivery [19]. Differences between the genders were also found pointing to higher productivity among female physicians particularly in primary care [15]. A recent study published in JAMA Internal Medicine found that older hospital patients treated by female internists had slightly lower death rates and readmission rates to the hospital, as compared to those treated by male physicians [20]. Although further research on differences such as these would need to be done in Israel, recognition of gender differences in workforce participation is important for policy-makers to consider in planning the development of the future healthcare workforce.

Since lifestyle issues regarding the balance between home and career are important in women’s deciding on amount of time to work in a week, choice of specialties etc., it is worthwhile to further study the use and effectiveness of incentives such as family-friendly working hours and training programs, childcare options and financial incentives when planning to attract female physicians to specific programs, specialties or geographic areas in Israel.

In Israel, as in other countries, age plays a role in how many hours doctors work, as well as how any more years they will continue to contribute to the workforce. Overall, fewer younger physicians than older ones worked less than full-time, with the percentage of physicians who worked shorter hours rising with age. A similar trend in European countries was seen wherein weekly work-hours rise for physicians between ages 35–39 and 45–49 but fall from 50 to 54 onwards as physicians approach retirement age [1]. Attempts to directly compare age characteristics between countries, however, is hampered when age and/or work-hour categories are not identical, as is often the case. An American Medical Association survey data from 2014 [18] did provide partially comparable data to our study, indicating that almost a quarter of US doctors worked 61 h or more, as compared to 15% of Israeli doctors.

The percentage of younger physicians in the labor pool in Israel has decreased over the years while doctors approaching retirement both comprised a growing segment of the labor force, and worked fewer hours [11]. In the United States as well, the age composition of the actively licensed physician population continues to display a definite shift from younger to older, reflecting the gradual but significant shift seen in the general population [21]. Studies from the U.S. have also shown that older physicians see fewer patients per day and work fewer weeks per year [1, 16], thus contributing even further to the diminishment of total physician care services. The Israel Medical Association (IMA) site reports that the average age of Israeli physicians is rising; in 1990, doctors under 45 constituted 48% of the physician population in Israel, in 2000 the percentage was 37%, and in 2008 only 27.7% [22] Our 2008-based data found that 10% of working physicians were aged 65 and over [3] and increased to almost 13% in 2012. In the US in 2010, a comprehensive analysis [21] found 22% of physicians to be 60 years or older, which increased to 26% in 2012 [23], identical to the 26% found in our current (2012) study.

The Horev committee reported on the aging of the medical specialist population based on data from the publication of the Health Information Department of the MoH [6]. In 1990 the rate of specialists under age 45 from among all specialists was 40% whereas in 2008 the rate was 26% and in 2012 it was 22.3%. The current study also illustrated that in some specialty fields young physicians were not replacing retirees at a compensatory rate. Furthermore, with Israel’s population growth, just replacing retiring physicians would not necessarily be adequate. This strengthens the argument that policy planners both encourage specialists, especially in fields of distress, to continue working past retirement age as well as incentivize the entry of young specialists into these fields [6, 13]. Even though older physicians predominantly work part-time, it is still likely to be beneficial to the healthcare system to have more physicians, especially those with much experience, working more years and retiring later.

The group responsible for the largest gap between the numbers of licensed and practicing physicians in Israel was that of physicians residing abroad. The findings indicated that at any point in time, approximately 10% of registered physicians of all ages were unavailable to the Israeli labor pool for this reason. The data also showed that the majority of those who were abroad were not young, recent licensees pursuing time-limited, post-graduate training. From among the 3275 physicians who were living abroad in 2012, 82% of them had been there for at least 5 years, a further indication that they may not have been there only temporarily.

The study could not provide information on whether the physicians residing abroad were actually employed, and if so, in what capacity, nor could it determine their reasons for moving abroad. However, the phenomenon of leaving the country, even if only for a period, in effect removed a portion of physicians from the employment pool in Israel. Seven percent of licensed younger physicians (aged 30–44) were abroad-- many perhaps pursuing advanced training with a plan to return to Israel upon completion. Other doctors will presumably take their places abroad as the former return. So, while it may be of benefit to the quality of the workforce to have its physicians pursue fellowships or sabbaticals abroad, for the purpose of workforce projection and planning, this absence needs to be taken into account.

We further recommend obtaining more information on Israeli physicians residing abroad to determine whether and in what capacity they are employed as well as any potential for their re-entry into the Israeli medical workforce and under what conditions. In addition, since our study shows the percentage abroad at a point in time, we recommend following this statistic over time.

Immigration out of a country may well be an indication of discontent, and could increase. Indeed, there is evidence of Israeli physicians’ lack of satisfaction, overload, burnout and consequent withdrawal from the profession [14, 15]. As one of its limitations, the current study could not directly shed light on the number of physicians who have abandoned the practice of medicine in favor of other areas of employment. A survey conducted by the Technion and published in 2003 [24] found that in retrospect, 12% of medical graduates would not have chosen to study medicine, an additional 23% were not sure whether they would have, and 4.2% of the physicians surveyed left medicine in favor of biotechnology or pharmaceutical industries and start-up companies. A later study published in 2011 [25] found that among young Israeli physicians licensed between the years 2000 and 2006, the drop-out rate from medicine among Israeli trained doctors was 5.6%, with another 10% having interviewed for jobs to examine their options for changing professions. It was also found that 13% of the physicians reported that they often contemplated the prospect of leaving the field, and only 65% of the sampled population of physicians never or very infrequently contemplated leaving. OECD international data [13] reported that about 5% - and the US Health Resources and Services Administration [26] reported about 6% - of licensed working age physicians were not active clinicians as their main occupation. In comparison, 1998 surveys from the United Kingdom found that as many as 17.6% to 21% of participants left medicine 7 to 11 years after graduation [27].

Reasons for abandoning the medical profession and doctors’ emigration are similar worldwide and include burnout, poor conditions in the country of origin including work conditions, attraction to other professions and the erosion of medical autonomy. For the most part, reasons for emigration and the abandonment of the field of medicine do not stem from doctors’ negative sentiments towards the profession itself, but from the conditions inherent in its practice [22]. The Van Dyke and Associates’ study [25] on Israel found a low level of satisfaction with the physical conditions of the work environment, the level of income and the lack of balance between work and personal life among medical practitioners. This is especially important as the field of medicine may be struggling to maintain its status in competition for the best undergraduate candidates among other lucrative professions such as high-tech and engineering.

We recommend that healthcare systems’ thinking about how to cope with existing or impending shortages in the supply of physicians, include both efforts for reduction on the demand side, as well as changes on the supply side. Doctors need to be supported to best leverage their time so that their days are not organized around tasks related to documentation and clerical and administrative activities which should be handled more appropriately by others in the system. Added clerical support can also reduce “no-shows” through concentrated appointment confirmations, especially in practices with limited or no clerical help, again, freeing up physician time to see more patients.

In a report of American experts independently addressing the question of how to meet the challenge of impending physician shortage, the most common thread running through their responses was that, to the greatest extent possible, other healthcare professionals should be working with physicians on multi-professional teams, with each member working "to the top of their license", and each member taking on tasks that match their professional competencies [28]. A team-based model of care is aspired to and exists to some extent in the Israeli healthcare system today. However, it may be achieved more thoroughly if and when inter-professional education is advanced, wherein clinicians who are initially trained in this approach become integrated into the system, as is recommended by these authors.

The team approach also incorporates the adoption of newer models of clinical care professionals which have been shown to be comparable to, or better than, physician-provided care on several process and outcome measures [29, 30]. In Israel in 2013, recommendations were accepted in the MoH regarding the expansion of clinical capacities of existing health professionals (nurses, paramedics etc.) in specific treatment areas so as to serve as physician assistants subordinate to and under the supervision of physicians [31]. At the end of 2013 the role of the nurse practitioner was created [32], authorizing the expansion of independent nursing responsibilities and treatments relevant to specific specialty areas such as palliative care, geriatrics, diabetes, neonatology, internal medicine, surgery and primary care.

Increasing the employment of such physician extenders in Israel, through both expanded training programs and broader acceptance and mobilization of these other professionals, could improve both access and quality of clinical care for patients. At the same time, it could free up physicians to do what only they are trained to do, and thus contribute to alleviating a potential physician shortage. Another specific recommendation that follows from the findings of the current research would be developing additional non-physician personnel such as clinical microbiology professionals and/or nurse anesthetists.

In addition, policymakers should consider promoting the use in Israel of specific technological advances, such as telemedicine for monitoring conditions remotely. This could result in reductions in use of services including fewer unnecessary physician visits and hospitalizations, thus easing up the demands on physicians’ time and enhancing efficiency [33].

The methodology of this study can serve as an important tool for policy planners to periodically provide up-to-date information on active healthcare workers and on those who are unavailable, either fully or partially, to the healthcare system, in the service of planning for a potential shortage [34]. To this end, it has been applied to similar studies on other health professions. Several such reports were published by the Ministry of Health in 2016 [35, 36] and others are in progress.

## Conclusions

Assessment of the medical workforce pool and personnel planning require not just the number of licensed physicians but also information about the employment mix of license holders and their level of professional activity in Israel.

Although a significant percentage of licensed Israeli physicians are living abroad at any point in time, major “brain drain” is not evident. For planning the numbers of physicians needed for the workforce, it is important to keep in mind that the average female vs. male physician has lower clinical productivity due to shorter hours and earlier retirement and that a group of young physicians will predictably be abroad at any point in time. Furthermore, extrapolating from the findings in the current studies, we believe that a potential shortage of physicians within Israel can be mitigated by better administrative support of physicians, use of physician extenders, and careful attention to improving physician satisfaction in certain specialties.

## Abbreviations

CBS: Central Bureau of Statistics
FTE: Full-time equivalent
IMA: Israel Medical Association
MoH: Ministry of Health
OECD: Organization for Economic Co-operation and Development
SEF: Socio-Economic File of the CBS Population Census Data

## Footnotes

1 A partial explanation for this phenomenon is that physicians who are employed by the army are not recorded in the income tax file. They comprise about 2.5% of all employed physicians in Israel though that is likely a higher percent among younger, male physicians, some of whom are engaged by the armed forces as part of an arrangement by which the army pays for their medical studies and they return professional service to the armed forces for 5 years upon completion of training.

2 Findings were based upon a literature review of online sources regarding re-registration practices in a selected group of western countries. See links, for some examples: https://www.mcnz.org.nz/maintain-registration/recertification-and-professional-development/

http://www.medicalboard.gov.au/Registration/Registration-Renewal.aspx

http://www.ct.gov/dph/cwp/view.asp?a=3121&q=443448
